# Sensing and Reliability Improvement of Electrostatic-Discharge Transient by Discrete Engineering for High-Voltage 60-V n-Channel Lateral-Diffused MOSFETs with Embedded Silicon-Controlled Rectifiers

**DOI:** 10.3390/s18103340

**Published:** 2018-10-06

**Authors:** Shen-Li Chen, Yi-Cih Wu

**Affiliations:** 1 Department of Electronic Engineering, National United University, Miaoli 36063, Taiwan; ilonawu514@gmail.com; 2 School of Information Engineering, Zhengzhou University, Zhengzhou 450066, China

**Keywords:** discrete modulation, electrostatic discharge (ESD), n-channel lateral-diffused MOSFET (nLDMOS), secondary breakdown current (*I_t2_*), silicon-controlled rectifier (SCR)

## Abstract

High-voltage n-channel lateral-diffused metal-oxide-semiconductor field-effect transistor (nLDMOS) components, fabricated by a TSMC 0.25-μm 60-V bipolar-CMOS-DMOS (BCD) process with drain-side embedded silicon-controlled rectifier (SCR) of the *n-p-n*-arranged and *p-n-p*-arranged types, were investigated, in order to determine the devices’ electrostatic discharge (ESD)-sensing behavior and capability by discrete anode engineering. As for the drain-side *n-p-n*-arranged type with discrete-anode manners, transmission–line–pulse (TLP) testing results showed that the ESD ability (*I_t2_* value) was slightly upgraded. When the discrete physical parameter was 91 rows, the optimal *I_t2_* reached 2.157 A (increasing 17.7% compared with the reference sample). On the other hand, the drain-side SCR *p-n-p*-arranged type with discrete-anode manner had excellent SCR behavior, and its *I_t2_* values could be increased to >7 A (increasing >281.9% compared with the reference DUT). Moreover, under discrete anode engineering, the drain-side SCR *n-p-n*-arranged and *p-n-p*-arranged types had clearly higher ESD ability, except for the few discrete physical parameters. Therefore, using the anode discrete engineering, the ESD dissipation ability of a high-voltage (HV) nLDMOS with drain-side SCRs will have greater effectiveness.

## 1. Introduction

Integrated-circuit (IC) technologies progress with each passing day. Trendily, the physical size of components decreases, the gate-oxide layer of transistors becomes thinner, and the junction depth becomes shallower. Consequently, smaller transistors are more vulnerable to the electrostatic-discharge (ESD) transient, and have a considerably higher failure rate [1,2,3,4,5,6]. The laterally-diffused metal-oxide-semiconductor field-effect transistor (LDMOSs) are often used in many integrated circuits of automotive electronics, power management circuits, power electronics, and communication modules [7,8,9,10,11,12] under high-voltage operation situations, owing to their distinguished characteristics, including being able to operate at a high blocking voltage and high conduction current. Because the device structure of a high-voltage (HV) LDMOS is complicated, designing an ESD protection unit into HV circuits is challenging [13,14,15,16,17,18,19]. ESD reliability is increasingly valued today—unfortunately, compared with low-voltage processes, HV processes have a lower robustness for ESD and electrical overstress (EOS) [20,21,22,23,24]. Thus, the design of HV ESD protection is worthy of innovation. This study addresses an HV nLDMOS as a fundamental structure with which to combine different drain-side SCR embedded architectures for improving ESD ability. A silicon-controlled rectifier (SCR) was built into the drain side, and discrete modulations were then employed on the anode region to even out the ESD transient current. In other words, this study utilized the discrete-anode method in the drain-side, thin-oxide definition (OD) region, together with a drain-side parasitic SCR, to strengthen the ability of the HV (60 V) nLDMOS to sense and resist the ESD pulse.

## 2. Testing Devices Layout

### 2.1. 60-V High-Voltage n-Channel Lateral-Diffused MOSFET Reference Device

**Figure 1:**
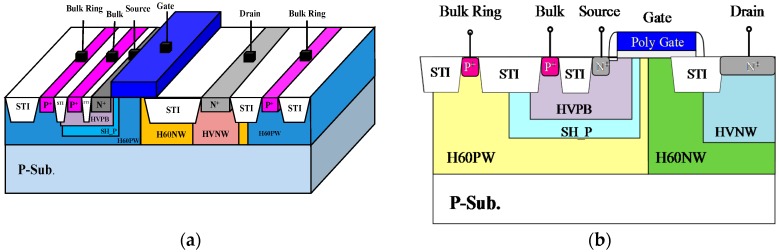
(**a**) Three-dimensional (3D) structure diagram, and (**b**) device cross-section view of an n-channel lateral-diffused MOSFET (nLDMOS) (reference device).

Conventional nLDMOSs often serve as ESD sensing and protection devices at the input or output ends of HV circuits because of their low on-resistance. However, nLDMOSs have an obvious weakness: they cannot be completely turned on in a multi-finger layout structure. Consequently, their ESD capability per unit width is very low, even at a very wide device width. Figure 1 shows the three-dimensional (3D) structure diagram and device cross-section view of the original HV nLDMOS (the reference device). The tested components were fabricated by a TSMC 60-V 0.25-μm bipolar-CMOS-DMOS (BCD) process. The source and bulk were with a non-butted structure, and the nLDMOS transistor had a multi-finger layout pattern. The channel length (*L*) was 2 µm, the width of every finger (*W_f_*) was 100 µm, and the finger number (M) of each transistor was 6, leading to a total channel width (*W_tot_*) of 600 µm.

### 2.2. 60-V High-Voltage n-Channel Lateral-Diffused MOSFET-Silicon-Controlled Rectifier: Anode-Discrete Modulations

**Figure 2:**
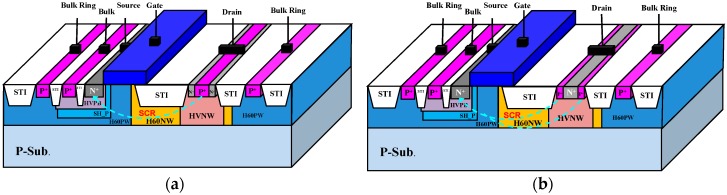
3D structure diagram of an nLDMOS-silicon-controlled rectifier (SCR) stripe: (**a**) *n-p-n*-arranged and (**b**) *p-n-p*-arranged.

The original wide-enough region on the drain side was used without adding any additional layout area for these HV LDMOSs with embedded SCRs. Meanwhile, from a circuit point of view, the electrical characteristics of this composite component can be changed little by appropriate optimization engineering, but the ESD reliability capability can be greatly improved. The drain side in an nLDMOS was planed in order to have three stripe zones. The zone-2 area was implanted with P^+^ dosages, which was equal to the sum of the zone-1 and -3 areas. Thus, an nLDMOS with an *n-p-n*-arranged SCR in the drain end was formed. Figure 2a shows the 3D structure of the HV nLDMOS-SCR drain-side stripe of the *n-p-n*-arranged type. In the same way, if the zone-1 and -3 areas were implanted with P^+^ dosages, but N^+^ dosages remained in the zone-2 area, and the areas sum of the zone-1 and -3 areas was equal to that of the zone-2 area, an nLDMOS with a *p-n-p*-arranged SCR in the drain end was fabricated. Figure 2b illustrates the 3D structure of the HV nLDMOS-SCR drain-side stripe *p-n-p*-arranged type.

**Figure 3:**
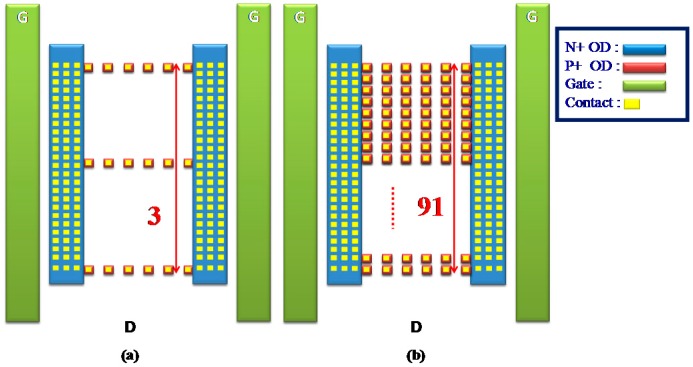
Schematic diagram of an nLDMOS-SCR (*n-p-n*-arranged and discrete-anode type): (**a**) DIS_3 and (**b**) DIS_91.

**Figure 4:**
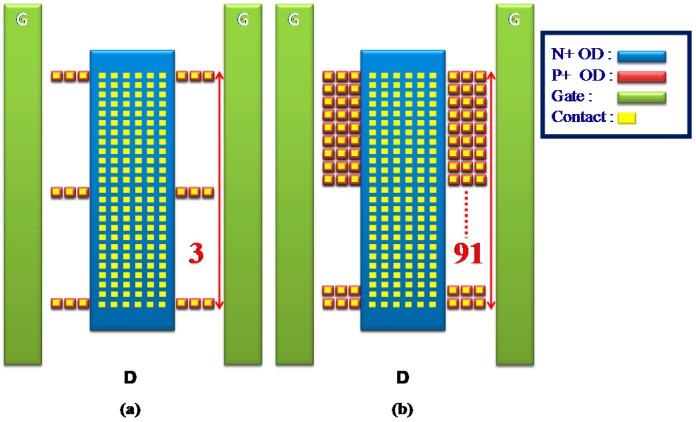
Schematic diagram of an nLDMOS-SCR (*p-n-p*-arranged and discrete-anode type): (**a**) DIS_3 and (**b**) DIS_91.

Commonly, a traditional SCR has a notable disadvantage: its *V_h_* value is very low. To improve the *V_h_* and solve the current non-uniform distributed problems, the zone-2 area (the anode) on the drain side only of an nLDMOS with an *n-p-n*-arranged SCR structure was discretized. Then, each thin oxide definition (OD) area was set at 0.7-μm in length and width, and its center fabricated with a contact measuring 0.3 μm in length and width. Every OD with its central contact was defined as a discrete contact, and each row had six discrete contacts for the *n-p-n*-arranged and *p-n-p*-arranged types. The discrete number at the marked end represents the discrete row number at the embedded SCR anode end (drain side). In other words, as the discrete number decreases, the proportion of OD at the SCR anode end decreases. Here, there are six sets of different groups for the SCR’s anode placement: 2, 3, 25, 47, 69, and 91. Figure 3a,b shows the schematics of the OD discrete groups DIS_3 and DIS_91 in an HV nLDMOS-SCR *n-p-n*-arranged type at the drain side. Similarly, Figure 4a,b shows the schematics of a 60-V HV nLDMOS-SCR *p-n-p*-arranged type at the drain side, with the OD discrete groups for the same parameters.

## 3. Transmission–Line–Pulse Testing Equipment

For experimental measurement, a transmission–line–pulse (TLP) testing system was applied and controlled by the LabVIEW environment platform. It manages the electronics subsystem and ultimately achieves the goal of fully automated testing. The output of this machine can provide a gradually increasing step-high voltage to a sample, and the short rising time for this square wave can also approximate the transient pulse of an ESD event. Then, this human-body model (HBM)-like equipment (TLP machine) can export testing waveforms with less than 10 ns rising/falling times and 100 ns pulse widths to evaluate the current-voltage (*I-V*) response of the DUT sample. Eventually, the precise ESD *I_t2_* value of the DUT is then obtained, and determined by whether the DUT leakage current is greater than 1 μA.

## 4. Measurement Results and Discussion

### 4.1. 60-V High-Voltage nLDMOS-Silicon-Controlled Rectifier: The Discrete n-p-n-Arranged Type

**Figure 5:**
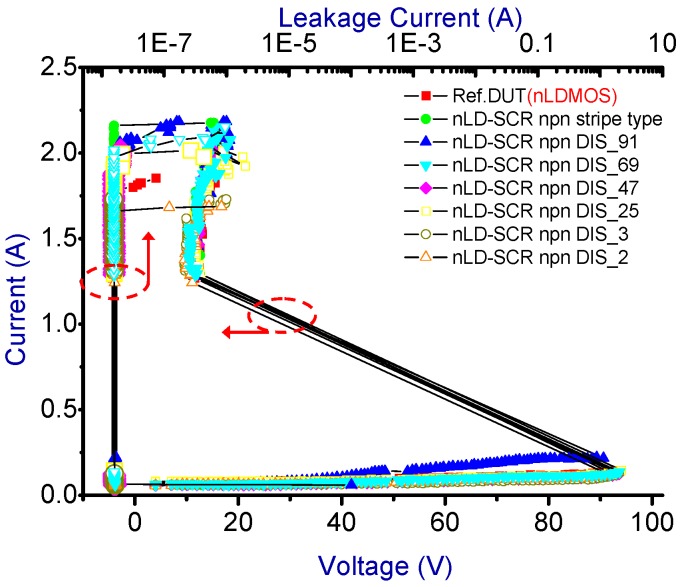
Snapback current-voltage curves and leakage currents of nLDMOS-SCR (*n-p-n*-arranged) tested samples.

**Figure 6:**
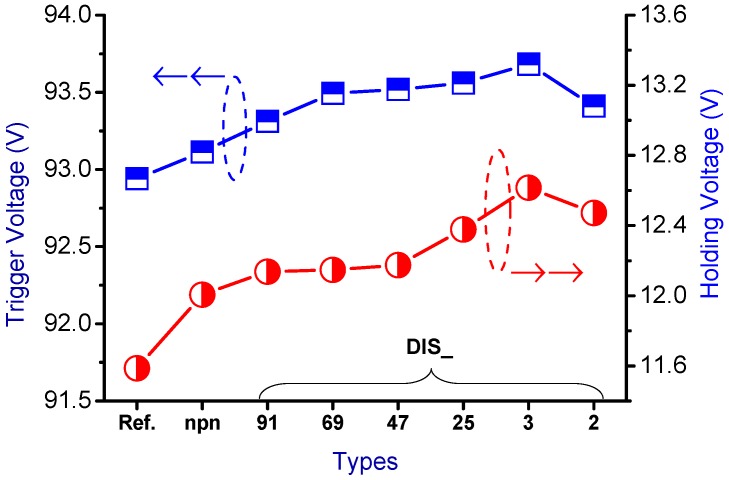
*V_t1_* and *V_h_* diagrams of the nLDMOS-SCR (*n-p-n*-arranged) tested samples.

The snapback *I-V* curves and corresponding physical parameters of the nLDMOS-SCR *n-p-n*-arranged type are presented in Figure 5 and Table 1. When the embedded SCR of the *n-p-n*-arranged type were inserted into the drain side of the nLDMOS, then the LDMOS and the embedded SCR form two parallel composite components. Since to the LDMOS drain-to-source distance is closer than that of the parasitic SCR anode to the cathode (source end) terminal, most of the ESD current flows to the LDMOS (the area occupied by this part became smaller compared to the reference DUT), and a small portion of ESD current was passed to the SCR. Consequently, the voltage had to be increased to trigger the component to conduct an ESD current. Therefore, the nLDMOS with the drain side-inserted SCR stripe *n-p-n*-arranged type required a higher trigger voltage (*V_t1_*) than the reference DUTs. When discrete modulation was applied to the anode end of the component’s drain-side parasitic SCR, the component’s *V_t1_* increased. The discrete method increased the SCR anode end’s parasitic resistance, which was the main reason for the increase in *V_t1_*. Therefore, except for the discrete parameter 2 (DIS_2; the smallest area of the SCRs), *V_t1_* increased as the discrete parameter was decreased. Figure 6 shows the trend in *V_t1_*. Meanwhile, the holding voltage (*V_h_*) was also related to on-resistance (*R_on_*). When the SCR’s anode end was discrete, as Figure 6 indicates, except for DIS_2, *V_h_* slowly and gradually increased as the discrete parameter was decreased.

**Figure 7:**
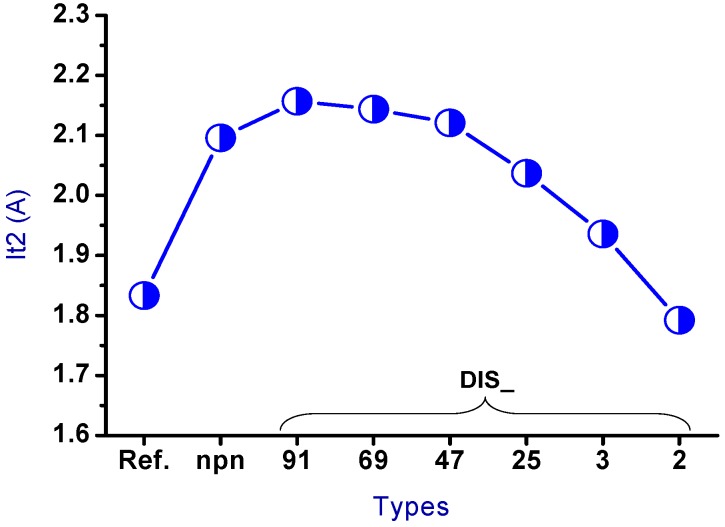
*I_t2_* diagrams of the nLDMOS-SCR (*n-p-n*-arranged) tested samples.

The trend for the secondary breakdown current (*I_t2_*) is shown in Figure 7. The drain side of the nLDMOS embedded with a stripe or discrete SCR *n-p-n*-arranged type formed a parallel composite circuit. Therefore, the *I_t2_* values of the stripe or discrete *n-p-n* arrangement were higher than the reference DUT, except for the DIS_2 sample. Moreover, the *I_t2_* values of nLDMOS-SCR discrete *n-p-n*-arranged type tends to decrease with the smaller discrete parameters, mainly due to the reduction of the area occupied by these embedded SCRs. For the discrete homogeneous DIS_91 sample, the *I_t2_* value is highest, and higher than that of the nLDMOS-SCR *n-p-n*-arranged stripe type sample (*I_t2_* = 2.096 A). This is due to the silicon substrate being a positive temperature coefficient material, and the discrete architecture helps to increase the conduction area. Therefore, the ESD (*I_t2_*) capability of DIS_91 has an optimum value of 2.157 A, which is 17.7% higher than the reference group *I_t2_* = 1.833 A.

### 4.2. 60-V High-Voltage nLDMOS-Silicon-Controlled Rectifier: The Discrete p-n-p-Arranged Type

**Figure 8:**
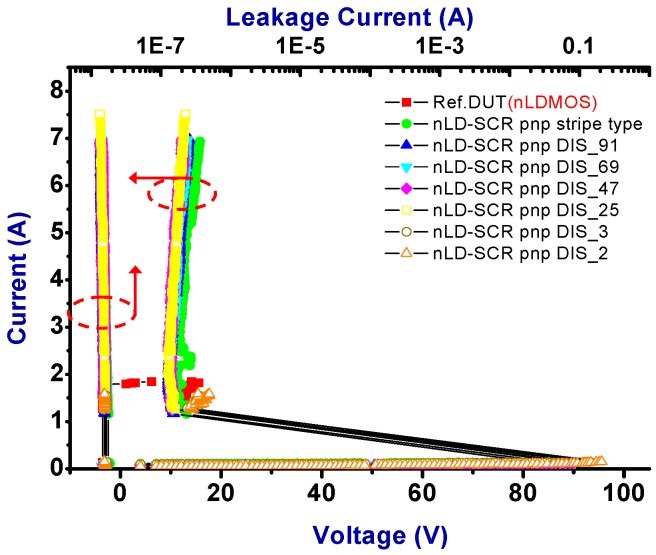
Snapback *I-V* curves and leakage currents of nLDMOS-SCR (*p-n-p*-arranged) tested samples.

**Figure 9:**
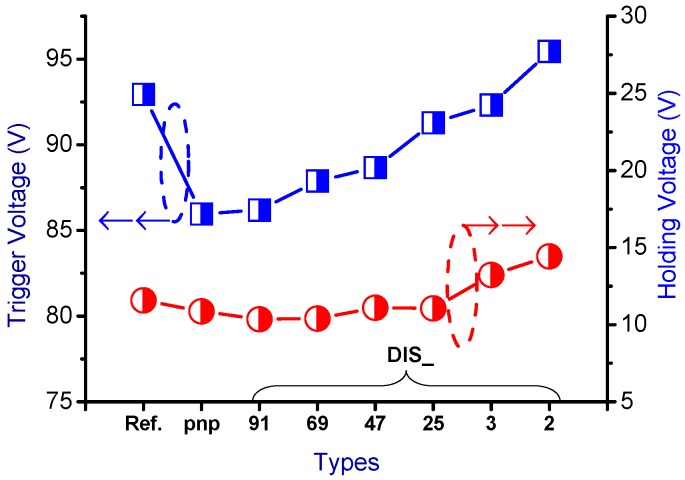
*V_t1_* and *V_h_* diagrams of the nLDMOS-SCR (*p-n-p*-arranged) tested samples.

**Figure 10:**
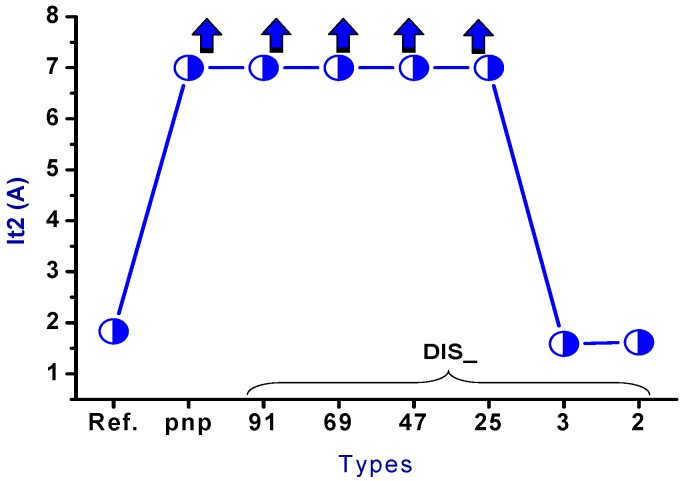
*I_t2_* diagrams of the nLDMOS-SCR (*p-n-p*-arranged) tested samples.

The snapback *I-V* curves and corresponding physical parameters of nLDMOS-SCR *p-n-p*-arranged type are presented in Figure 8 and Table 2. Similarly, when the embedded SCR of the *p-n-p*-arranged type was inserted into the drain side of the nLDMOS, then the LDMOS and the embedded SCR formed two parallel composite components. It can be seen from Figure 8 and Table 2 that the nLDMOS with a drain-side parasitic SCR of the *p-n-p*-arranged type has obvious SCR characteristics, whether it is the stripe or discrete type. This is because the SCR conduction path in the *p-n-p*-arranged type from the anode terminal to the cathode (source end) is shorter than the conduction path of the nLDMOS—most of the ESD current flows to the SCR. Therefore, there is a very strong ESD (*I_t2_*) capacity per unit width for these samples. In addition, the HV SCR has a lower on-resistance than the same-process LDMOS; the trigger voltage (*V_t1_*) of the *p-n-p*-arranged stripe type will be lower than that of the reference nLDMOS [25]. This study also discovered that when the anode end of an nLDMOS-SCR in the *p-n-p*-arranged type was discretely modulated, the component’s *V_t1_* increased. However, the *V_t1_* and *V_h_* values increased as the discrete number decreased in the *p-n-p*-arranged discrete type, as shown in Figure 9. Here, *V_h_* was related to *R_on_*—as the SCR’s anode end was discrete, *V_h_* values slowly and gradually increased. Finally, the ESD (*I_t2_*) capability of the nLDMOS-SCR *p-n-p*-arranged type is shown in Figure 10; *I_t2_* values are higher than 7 A (due to the power limitation of the TLP testing system, measurement was stopped when the current of DUTs was >7 A), except for the DIS_3 and DIS_2 parameters (SCR conduction areas were too small).

### 4.3. High-Voltage nLDMOS and nLDMOS-Silicon-Controlled Rectifier TCAD Simulation and Verification

**Figure 11:**
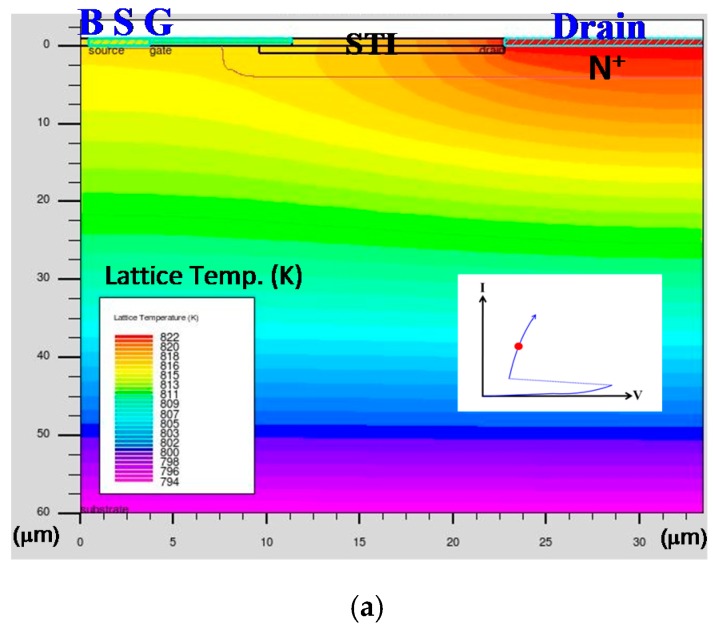

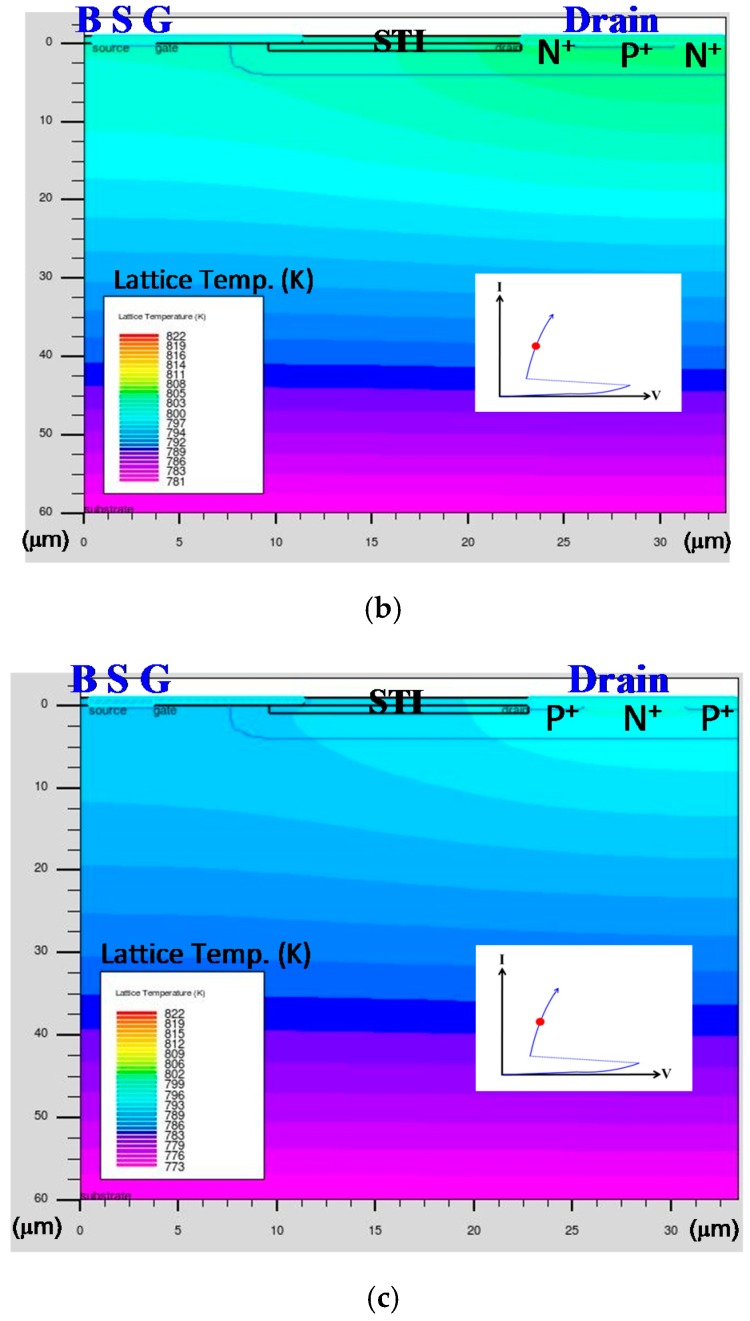
Lattice temperatures distribution of 60 V HV nLDMOS (**a**) reference device, (**b**) drain-side *n-p-n*-arranged type, and (**c**) drain-side *p-n-p*-arranged type stressing at the same trigger condition.

The value of the *I_t2_* component is actually the maximum current that can be conducted before the component reaching the physical melting point—that is, the last moment before the component is destroyed. Therefore, the lattice temperature of a DUT in a simulation is related to the ESD capability value. If a component’s lattice temperature is higher as the bias conditions in simulation are kept the same, it means that this tested sample will be less resistant to a high ESD current. Figure 11 presents the lattice temperature distribution of 60-V nLDMOS devices by the (a) reference DUT, (b) drain-side parasitic SCR *n-p-n*-arranged type, and (c) drain-side parasitic SCR *p-n-p*-arranged type. Clearly, the parasitic SCR structures in Figure 11b,c had low on-resistance under the same drain trigger conditions, and thus had the characteristic of low power consumption (with more lower-lattice-temperature distributed profiles), and eventually they could discharge higher ESD currents. For Figure 11c especially, the nLDMOS with a drain-side parasitic SCR *p-n-p*-arranged type has a shortest conduction path for the SCR, with obvious SCR characteristics. In other words, it is found from the lattice temperature distribution diagram of Figure 11a that the reference sample has higher lattice temperature profile than the *n-p-n*-type or the *p-n-p*-type, and the high lattice temperature regions will concentrate on the entire drain region. As shown in Figure 11b,c, it is found that the lattice temperature distribution of the *p-n-p*-type is lower lattice temperature profiles over the entire flow path than the *n-p-n*-type. Unlike the *n-p-n*-type high lattice-temperature profile, the *p-n-p*-type profile has a lower lattice temperature distribution in the drain region. Therefore, the TCAD simulation results also confirmed that an nLDMOS parasitic SCR with a *p-n-p*-arranged type will more effectively enable the protective component, in order to discharge the ESD current.

## 5. Conclusions

HV nLDMOS drain-side embedded SCR *n-p-n*- and *p-n-p-*arranged types, whether strip or discrete type (except for the small discrete number groups), can significantly improve the ESD (*I_t2_*) value. Among these, the drain-side embedded SCR *p-n-p-*arranged type has strong SCR characteristics, and the ESD robustness is excellent (*I_t2_* > 7 A); for the *n-p-n-*arranged type, the *I_t2_* value has an optimal value of 2.157 A (at the same group), as the discrete parameter is 91, which is higher than the corresponding *n-p-n* strip type and reference DUTs. In addition, the parasitic SCR at the drain end causes a slight decrease in *V_h_*, especially for the *p-n-p-*arranged type. Generally, the stronger the SCR characteristics, the more significant in the *V_h_* decreasing. However, the holding voltage (*V_h_*) generally increases as the number of OD discrete groups decreases (and the on-resistance (*R_on_*) increases). Finally, it was verified by the TCAD simulation results that the drain-side parasitic SCR structure (especially for the embedded SCR *p-n-p-*arranged type) has a more uniform and deeper conduction path under the same triggering conditions than the *n-p-n-*arranged type. Then, combined with low power consumption, it allows for higher ESD dissipative currents.

## Figures and Tables

**Table 1:** Snapback key parameters of the nLDMOS-SCR (*n-p-n*-arranged) tested samples.

nLDMOS + Drain SCR		*V_t1_* (V)	*V_h_* (V)	*I_t2_* (A) (mean ± σ)
**Ref. DUT (nLDMOS)**		92.941	11.587	1.833 ± 0.083
**nLD-SCR npn Stripe type**		93.114	12.006	2.096 ± 0.214
***n-p-n*-type**	**DIS_91**	93.313	12.138	2.157 ± 0.034
	**DIS_69**	93.494	12.148	2.144 ± 0.078
	**DIS_47**	93.518	12.175	2.121 ± 0.163
	**DIS_25**	93.559	12.378	2.034 ± 0.019
	**DIS_3**	93.683	12.616	1.936 ± 0.066
	**DIS_2**	93.41	12.472	1.792 ± 0.024

**Table 2:** Snapback key parameters of the nLDMOS-SCR (*p-n-p*-arranged) tested samples.

nLDMOS + Drain SCR		*V_t1_* (V)	*V_h_* (V)	*I_t2_* (A) (mean ± σ)
**Ref. DUT (nLDMOS)**		92.941	11.587	1.833±0.083
**nLD-SCR pnp Stripe type**		85.971	10.863	>7
***p-n-p*-type**	**DIS_91**	86.189	10.358	>7
	**DIS_69**	87.889	10.401	>
	**DIS_47**	88.661	11.106	>7
	**DIS_25**	91.287	11.051	>7
	**DIS_3**	92.326	13.220	1.59 ± 0.2
	**DIS_2**	95.445	14.434	1.618 ± 0.084
